# Attitudes and Response Capacities for Public Health Emergencies of Healthcare Workers in Primary Healthcare Institutions: A Cross-Sectional Investigation Conducted in Wuhan, China, in 2020

**DOI:** 10.3390/ijerph191912204

**Published:** 2022-09-26

**Authors:** Changmin Tang, Xin Chen, Cuiling Guan, Pengqian Fang

**Affiliations:** 1 School of Management, Hubei University of Chinese Medicine, Wuhan 430065, China; 2 Hubei Provincial Key Research Base of Humanities and Social Sciences, Wuhan 430065, China; 3 Tongji Medical College, Huazhong University of Science and Technology, Wuhan 430030, China

**Keywords:** attitude, response capacities, public health emergencies, healthcare workers, primary healthcare institutions

## Abstract

Objectives: Response capacities for public health emergencies (PHEs) amongst healthcare workers play important roles in the prevention and control of PHEs. This study assessed the attitudes and response capacities of PHE workers in primary healthcare (PHC) institutions. Methods: An online anonymous questionnaire survey of 803 healthcare workers sampled from 13 PHC institutions in Wuhan, China, was conducted from April to June 2020. The Kruskal–Wallis test and linear regression model were used to analyze the response capacities of PHE workers and associated factors. Results: The healthcare workers with longer working years, particularly 30 years and above, had higher knowledge (OR = 7.323, *p* < 0.001) and practical ability scores (OR = 8.012, *p* < 0.001) when compared to those with less than 5 working years. The nurses had higher practical ability scores (OR = 2.188, *p* = 0.049), and pharmacists had lower practical ability scores (OR = 0.166, *p* = 0.007), when compared to doctors. Moreover, the healthcare workers who had never participated in educational activities related to PHE management in the past two years (OR = 0.540, *p* = 0.038; OR = 0.282, *p* = 0.001), had not participated in a PHE drill activity (OR = 0.327, *p* < 0.001; OR = 0.340, *p* = 0.004), and had never been involved in emergency management of PHEs (OR = 0.254, *p* < 0.001; OR = 0.174, *p* < 0.001) had lower knowledge and practical ability scores. Conclusion: The healthcare workers with longer working years had better response capacities, and nurses had better practical abilities when compared to doctors. More emergency management education and chances to be involved in PHE drill activities were encouraged amongst healthcare workers in PHC institutions for better prevention and control of PHEs. Moreover, inter-institution cooperation, a flexible response system, and dynamic adjustment of healthcare workers were suggested during PHEs.

## 1. Background

Public health emergencies (PHEs) have important impacts on the population’s health as well as on the development of the global economy [1]. Ever since the SARS epidemic, and considering its impact on global health and the economy in 2002, PHEs of international concern have been focused on by the World Health Organization to reduce the effects on global health and the economy [2]. PHEs refer to major infectious disease epidemics, mass unexplained diseases, major food and occupational poisoning, including other events that occur suddenly, and cause (or may cause) serious damage to public health. In addition, the major characteristics of PHEs are suddenness, publicity, and urgency [3]. Timely and effective emergency response capabilities are considered important measures to control emergency incidents. Emergency management of PHE(s) (EMPHE) is receiving more attention in many countries, including China. Emergency management assessments are also considered important works in China [4]. Furthermore, the coronavirus disease 2019 (COVID-19) outbreak highlights the importance of EMPHE.

Medical institutions are the main institutions that respond to PHEs, carrying out emergency treatments and controls. Primary healthcare (PHC) has played an important role in the health service system, providing basic public health services, such as prevention, basic healthcare (common and frequently occurring diseases), rehabilitation, and health education for residents in China [5,6]. In the prevention and control of the COVID-19 epidemic in 2020, PHC played an important role in community prevention and control. Moreover, the construction of a grass-roots public health system is an important part of the construction of a strong public health service system. From January to March 2020, the National Health Commission of the People’s Republic of China issued a series of policies to further clarify the important roles of PHC institutions in the prevention and control of the COVID-19 epidemic. Examples include ‘Notice on Strengthening the Prevention and Control of Novel Coronavirus Pneumonia Epidemic in Primary Healthcare Institutions’ and ‘Notice on the Classification and Accurate Work of Primary Healthcare Institutions in the Prevention and Control of Novel Coronavirus Pneumonia Epidemic’. These policies include requirements for PHC institutions to conduct good jobs in the prevention and control of COVID-19.

In addition, the response capacities of PHE workers in medical institutions and PHC institutions play critical roles in emergency response work [7]. Response capacities and the professional levels of healthcare workers directly affect the implementation of epidemic prevention and control [8]. In terms of the literature analysis, knowledge, attitude(s), and practice(s) (KAP) have been used in some studies to analyze the response capacities of PHE workers, particularly toward COVID-19 [9,10]. Moreover, some studies have analyzed the response capacities of healthcare workers in community health centers toward COVID-19 in China [11]. However, studies analyzing the response capacities of PHE workers in PHC institutions in China with KAP are limited.

In the prevention and control of COVID-19 in 2020, healthcare workers in Wuhan endured extraordinary pressures and were regarded as heroes who protected the health of the Chinese people and the people across the world. The people and healthcare workers of Wuhan made great sacrifices in the fight against COVID-19 and made significant contributions to the prevention and control of COVID-19. They also bought precious time for the prevention and control of the epidemic, nationally and globally. However, some insufficiencies should be improved.

This study analyzed the attitudes and response capacities (including knowledge and practical abilities) for PHE of healthcare workers in PHC institutions after the COVID-19 outbreak in Wuhan, China.

## 2. Methods

### 2.1. Study Design

The cross-sectional study was conducted in Wuhan, Hubei Province, China, from April to June 2020, after the city was locked down. Wuhan, the capital city of Hubei Province, located in the central area of China, was critically affected by COVID-19. As Wuhan has 13 administrative regions, one PHC institution was selected from each region using the convenience sampling method in our survey. A total of 13 PHC institutions (including 7 community health centers in the urban area and 6 township health centers in the suburban area) agreed to conduct the survey. All healthcare workers in PHC institutions were asked to complete the questionnaire with their consent.

### 2.2. Questionnaire

A self-designed anonymous questionnaire was used to collect the data, which included the following: (1) the characteristics of the healthcare workers surveyed (including gender, working years, highest education, profession, and institution); (2) previous experiences of the healthcare workers regarding training, drills, and participation in PHE management; (3) attitudes of medical workers in PHC institutions toward PHEs (including nine items); (4) response capacities of PHE workers (including knowledge (four items) and practical ability (five items) listed in Tables S1 and S2 in the Supplementary Materials). Attitude and response capacities were derived from the relevant literature research [12,13]. Then, through an internal seminar of the research group, the items were adjusted. The answers to the three items concerning previous experiences were set as yes or no. Nine items measuring attitude and nine items measuring response capacities were measured using a five-point Likert scale, ranging from 1 ‘strongly disagree’ to 5 ‘strongly agree’.

### 2.3. Data Collection

An online questionnaire was sent to each institution, and the healthcare workers were asked to fill it out with the help of managers. On average, each survey took approximately five minutes to complete, and each participant could only submit the questionnaire once. Implied informed consent was obtained from each participant prior to the survey. A total of 803 healthcare workers filled out the questionnaire, and the data were used for analysis. Cronbach’s alpha coefficient of attitude (nine items), knowledge (four items), and practical ability (five items) were 0.947, 0.951, and 0.951, respectively. Moreover, Cronbach’s alpha coefficient of response capacity (nine items, including knowledge and practical ability) was 0.966, which indicated a good internal consistency.

### 2.4. Statistical Analysis

The sociodemographic characteristics of respondents and previous experiences of the healthcare workers regarding training, drills, and participation in PHE management were described through frequency distributions. Moreover, the attitude and response capacities (including knowledge and practical ability) of healthcare workers surveyed on PHEs were described through mean and variance. The Kruskal–Wallis test and linear regression model were used to analyze the response capacities of PHE workers and associated factors. Statistical significance was set at *p* < 0.05 for the Kruskal–Wallis test and the linear regression model analysis.

## 3. Results

### 3.1. Characteristics of the Healthcare Workers Surveyed

Of the total healthcare workers surveyed, 21.9% were male, and 78.1% were female. Most of the respondents worked between 5 and 29 years, i.e., 73.5% and 26.0% of them worked between 20 and 29 years. Regarding the highest level of education achieved, 49.2% of respondents were in junior college or below, 47.9% were undergraduates, and just 2.9% were graduates. Moreover, 32.1% of the respondents were doctors, 39.5% were nurses, 12.3% were public health personnel, and 16.1% were other healthcare workers. Moreover, 50.1% of the respondents were located in community health centers, and 49.9% were in township health centers (Table 1).

### 3.2. Attitudes of Healthcare Workers toward PHEs

Of the healthcare workers surveyed, the highest score on the attitude item was that PHC institutions should formulate relevant strategies and plans for joint different institutions to respond to PHEs (4.71 ± 0.618); 79.1% strongly agreed with this. Table 2 presents the rest of the attitudes toward PHE workers in PHC institutions.

### 3.3. Previous Experience of Healthcare Workers concerning PHEs

Of the healthcare workers surveyed, 70.2% had participated in educational activities related to EMPHE over the past two years. Moreover, 60.0% had participated in a PHE drill activity, and 45.2% had been involved in EMPHEs (Table 3).

### 3.4. Response Capacities and Associated Factors Based on the Kruskal–Wallis Test

Through the Kruskal–Wallis test analysis (Table 4), the healthcare workers with longer working years (*p* < 0.001, *p* < 0.001) and the lower highest education (*p* < 0.001, *p* < 0.001), and located in the township health center (*p* = 0.014, *p* = 0.001), had higher knowledge and practical ability scores. Moreover, the nurses had higher practical ability scores when compared with other professions (*p* = 0.001). The healthcare workers who had participated in educational activities (related to PHE management over the past two years) had participated in a PHE drill activity, and had been involved in EMPHEs had higher knowledge and practical ability scores (*p* < 0.001).

### 3.5. Response Capacities and Associated Factors Based on the Linear Regression Model

Furthermore, through the linear regression model analysis (Table 5 and Table 6), the healthcare workers with longer working years, particularly 30 years and above, had higher knowledge (OR = 7.323, *p* < 0.001) and practical ability scores (OR = 8.012, *p* < 0.001) when compared with those with less than 5 working years. The healthcare workers with graduate (OR = 0.128, *p* = 0.003; OR = 0.176, *p* = 0.049) and undergraduate degrees (OR = 0.368, *p* < 0.001; OR = 0.372, *p* = 0.001) had lower knowledge and practical ability scores when compared with the healthcare workers whose education consisted of junior college or below. The nurses had higher practical ability scores (OR = 2.188, *p* = 0.049), and pharmacists had lower practical ability scores (OR = 0.166, *p* = 0.007), when compared to doctors.

Moreover, the healthcare workers who had never participated in educational activities related to PHE management over the past two years (OR = 0.540, *p* = 0.038; OR = 0.282, *p* = 0.001), had not participated in a PHE drill activity (OR = 0.327, *p* < 0.001; OR = 0.340, *p* = 0.004), and had never been involved in EMPHEs (OR = 0.254, *p* < 0.001; OR = 0.174, *p* < 0.001) had lower knowledge and practical ability scores.

### 3.6. Correlation Amongst Attitude, Knowledge, and Practical Ability

Through a Pearson correlation analysis of attitude, knowledge, and practical abilities (Table 7), a significant correlation was found (*p* < 0.001). Attitude had significant correlations with the knowledge and practical abilities of healthcare workers in PHC institutions; moreover, knowledge had a significant correlation with practical abilities.

## 4. Discussion

From our survey and data analysis, we found that the response capacities of healthcare workers in PHC institutions were associated significantly with working years and the highest education. Healthcare workers who worked more years and had ‘lower’ highest education types had higher knowledge and practical ability scores. This result may be surprising, i.e., why would a healthcare worker with lower education have higher knowledge and a practical ability score? One possible reason is that healthcare workers with longer working years had lower education levels in community health centers and township health centers. According to China’s Health Statistical Yearbook, only 12% of healthcare workers had undergraduate degrees or above in community health centers in 2005, 18.4% in 2010, and 43.2% in 2020. Moreover, only 2.2% of healthcare workers had undergraduate degrees or above in township health centers in 2005, 5.7% in 2010, and 22.2% in 2020 in China [14,15]. Moreover, ‘working years’ was a very important factor influencing the knowledge and practical abilities of healthcare workers, and the results were similar to other studies [16,17].

In terms of profession, when compared to doctors, nurses had higher practical PHE ability scores, and pharmacists had lower scores in the study. Compared to doctors, the nurses in PHC institutions conducted more basic work in response to PHEs, such as nucleic acid testing and so on, and cooperated and communicated with the community more in order to perform the joint prevention and control mechanisms well in China, which might have enabled nurses who had higher practical ability scores for PHEs. Moreover, we observed some similar results in previous studies, which showed that nurses’ emergency response abilities were higher than those of doctors during the COVID-19 period [18]. Paramedics (not including doctors and nurses) were also found to be less likely to wash their hands frequently when compared to doctors [9]. In Saudi Arabia, nursing staff workers were more likely to comply with appropriate infection prevention and control practices when compared with medical or surgical staff and pharmacy staff [19]. Therefore, the practical ability and response capacities of doctors in PHC institutions should be improved as frontline healthcare workers play important roles in the prevention and control of PHEs.

In addition, 70.2% of the healthcare workers surveyed had participated in educational activities related to EMPHE over the past two years, which was strongly associated with the response capacities of healthcare workers (including knowledge and practical ability, *p* < 0.05). The healthcare workers who had participated in educational activities had higher knowledge and practical ability scores in the study. PHE education and training had important impacts on response capacities, which had been researched in many studies [20,21,22]. However, studies have paid more attention to emergency management education and PHE training of leaders [23]. The related education and training were also important amongst healthcare workers in the analysis so they could understand the whole management, more clearly see what they could do, and understand the value of the prevention and control of PHEs.

Moreover, 45.2% of the healthcare workers surveyed had been involved in EMPHE, which was significantly associated with the response capacities of healthcare workers (including knowledge and practical ability, *p* < 0.001). As discussed above, healthcare workers could be involved in EMPHE, which could help improve their response capacities. Emergency healthcare worker preparedness for disaster management should be focused on more [24], and providing more chances to healthcare workers to be involved in EMPHE is also suggested.

Disaster and PHE drills played important roles in emergency preparedness and in improving the rapid response, organizational coordination, and emergency response capabilities of relevant institutions [25,26,27]. Moreover, drills are a great way to provide education and training [28]. A PHE drill is also an important means to test the construction of the health emergency system, emergency plan, emergency response, emergency rescue capabilities, emergency management, and other factors. In the study, 60.0% of the healthcare workers surveyed had participated in PHE drill activities, which also significantly influenced the response capacities of healthcare workers (including knowledge and practical ability, *p* < 0.01). Although the percentage was higher than in some other studies before the COVID-19 outbreak [25,29], a PHE drill received more attention in many countries, including China, as a global threat to health and the economy. This study confirmed that more PHE drills could improve the response capacities of healthcare workers. Therefore, more PHE drills are encouraged amongst healthcare workers in PHC institutions, and every healthcare worker should have the opportunity to participate in the drills, if possible.

Therefore, as discussed above, we should pay more attention to improving the practical abilities and response capacities of doctors in PHC institutions. Strengthening emergency management is beneficial in improving the overall emergency management efficiencies of PHEs [30]. Hence, more emergency management education and chances to be involved in PHE emergency management were encouraged amongst healthcare workers. Emergency management education could be arranged in a variety of ways, including online. We could increase PHE management education and opportunities for healthcare workers by introducing it into their regular work schedules, according to the requirement surveys of healthcare workers, and design reasonable ways to improve the response capacities of PHEs. In addition, more disaster and PHE drills are suggested for healthcare workers in PHC institutions. Regarding the attitudes of healthcare workers toward PHEs—inter-institution cooperation, a flexible response system, and dynamic adjustment of health human resources amongst different institutions were suggested during PHEs [31].

This study has some limitations. Firstly, the cross-sectional investigation of healthcare workers was conducted after the 2020 COVID-19 outbreak in Wuhan, rather than being conducted with comparisons at later stages. If possible, we hope to obtain further research. Secondly, a self-designed online questionnaire in Chinese was used to collect the data, and limited items were designed according to the relevant literature research and internal seminar of the research group. Thirdly, the investigation was only conducted in Wuhan, Hubei Province, China.

## 5. Conclusions

Healthcare workers with longer working years had better response capacities (including knowledge and practical abilities). Nurses had higher practical ability scores for PHEs, and pharmacists had lower scores when compared to doctors. Emergency management education, involvement in emergency management, and participation in PHE drill activities were significantly associated with the response capacities of healthcare workers. Emergency management education, chances involved in emergency management, and PHE drill activities were encouraged amongst healthcare workers in PHC institutions to better prevent and control PHEs. Furthermore, inter-institution cooperation, a flexible response system, and the dynamic adjustment of healthcare workers were suggested during PHEs.

## Figures and Tables

**Table 1:** Characteristics of the healthcare workers surveyed (N = 803).

Characteristics	N	%
Gender		
Male	176	21.9
Female	627	78.1
Working years		
Less than 5 years	133	16.6
5–9	193	24.0
10–19	189	23.5
20–29	209	26.0
30 years and above	79	9.8
Highest education		
Junior college or below	395	49.2
Undergraduate	385	47.9
Graduate	23	2.9
Profession		
Doctor	258	32.1
Nurse	317	39.5
Public health personnel	99	12.3
Pharmacist	41	5.1
Inspector	39	4.9
Other healthcare workers	49	6.1
Institutions		
Community health center	402	50.1
Township health center	401	49.9
Total	803	100

**Table 2:** Attitudes of healthcare workers at PHC institutions (toward PHEs).

Items	Strongly Agree (N/%)	Mean	SD
1. Primary healthcare (PHC) institutions should have emergency management procedures and rules on public health emergencies (PHEs).	625 (77.8)	4.69	0.659
2. PHC institutions should formulate relevant strategies and plans for different institutions to respond to PHEs.	635 (79.1)	4.71	0.618
3. PHC institutions need to regularly review and update institutional strategies for emergency responses to PHEs.	606 (75.5)	4.66	0.678
4. All healthcare workers in PHC institutions should be familiar with institutional strategies for implementing EMPHEs.	619 (77.1)	4.70	0.602
5. All healthcare workers in PHC institutions should be familiar with the identification process for PHEs and should have knowledge of how to implement the necessary procedures.	611 (76.1)	4.69	0.607
6. All healthcare workers in PHC institutions must receive education and training in emergency response to PHEs.	624 (77.7)	4.69	0.635
7. The overall and situational risk awareness of PHEs needs to be high amongst healthcare workers in PHC institutions.	599 (74.6)	4.66	0.647
8. The work content of PHC institutions in daily operations and PHEs should be adjusted.	553 (68.9)	4.58	0.686
9. PHC institutions should frequently conduct relevant drills to effectively conduct emergency management in the event of PHEs.	580 (72.2)	4.62	0.686

**Table 3:** Previous experience of the healthcare workers regarding training, drills, and participation in PHE management (N = 803).

Items	Yes (N/%)	No (N/%)
Over the past two years, have you participated in any educational activities related to EMPHE?	564 (70.2)	239 (29.8)
Have you ever participated in any PHE drill activity?	482 (60.0)	321 (40.0)
Have you ever been involved in EMPHE?	363 (45.2)	440 (54.8)

**Table 4:** Response capacities for PHEs of healthcare workers and associated factors (Kruskal–Wallis test).

Characteristics	Knowledge			Practical Ability		
	Mean ± SD	F	*p*	Mean ± SD	F	*p*
Gender		3.715	0.054		3.001	0.083
Male	17.40 ± 3.28			21.29 ± 4.42		
Female	16.82 ± 3.37			20.76 ± 4.30		
Working years		35.720	<0.001		23.802	<0.001
Less than 5 years	15.72 ± 3.65			19.70 ± 4.76		
5–9	16.80 ± 3.29			20.93 ± 4.34		
10–19	16.96 ± 3.38			20.63 ± 4.42		
20–29	17.28 ± 3.08			21.11 ± 4.01		
30 years and above	18.48 ± 2.91			22.66 ± 3.52		
Highest education		34.834	<0.001		27.403	<0.001
Junior college or below	17.61 ± 3.16			21.61 ± 4.12		
Undergraduate	16.38 ± 3.45			20.23 ± 4.47		
Graduate	15.26 ± 2.85			19.13 ± 3.39		
Profession		8.849	0.115		20.725	0.001
Doctor	16.79 ± 3.29			20.50 ± 4.37		
Nurse	17.36 ± 3.15			21.75 ± 3.78		
Public health personnel	16.63 ± 3.58			20.02 ± 4.81		
Pharmacist	16.95 ± 3.79			19.83 ± 5.63		
Inspector	16.31 ± 3.74			20.59 ± 4.13		
Other healthcare workers	16.31 ± 3.63			19.98 ± 4.53		
Institutions		6.065	0.014		10.356	0.001
Community health center	16.65 ± 3.37			20.40 ± 4.39		
Township health center	17.25 ± 3.31			21.35 ± 4.22		
Educational activities		52.924	<0.001		70.435	<0.001
Yes	17.57 ± 2.94			21.75 ± 3.87		
No	15.48 ± 3.79			18.81 ± 4.65		
Drill activities		86.049	<0.001		88.862	<0.001
Yes	17.87 ± 2.79			22.04 ± 3.83		
No	15.56 ± 3.65			19.13 ± 4.45		
Emergency management		104.454	<0.001		105.928	<0.001
Yes	18.24 ± 2.71			22.55 ± 3.60		
No	15.89 ± 3.46			19.50 ± 4.40		
Total	16.95 ± 3.35			20.88 ± 4.33		

**Table 5:** Knowledge of healthcare workers toward PHEs and associated factors (linear regression model).

Characteristics	B	*p*-Value	95% Confidence	OR
Gender				
Men				
Women	−0.300	0.324	(−0.898, 0.297)	0.741
Working years				
Less than 5 years				
5–9	1.156	0.001	(0.483, 1.829)	3.177
10–19	1.303	<0.001	(0.627, 1.979)	3.680
20–29	1.373	<0.001	(0.683, 2.064)	3.947
30 years and above	1.991	<0.001	(1.104, 2.878)	7.323
Highest education				
Junior college or below				
Undergraduate	−0.999	<0.001	(−1.470, −0.528)	0.368
Graduate	−2.053	0.003	(−3.391, −0.715)	0.128
Profession				
Doctor				
Nurse	0.107	0.728	(−0.498, 0.712)	1.113
Public health personnel	−0.468	0.217	(−1.213, 0.276)	0.626
Pharmacist	−0.812	0.114	(−1.819, 0.194)	0.444
Inspector	−0.162	0.761	(−1.203, 0.880)	0.850
Other healthcare workers	−0.172	0.717	(−1.101, 0.757)	0.842
Institutions				
Community health center				
Township health center	−0.021	0.929	(−0.482, 0.440)	0.979
Educational activities				
Yes				
No	−0.616	0.038	(−1.197, −0.035)	0.540
Drill activities				
Yes				
No	−1.117	<0.001	(−1.692, −0.541)	0.327
Emergency management				
Yes				
No	−1.370	<0.001	(−1.883, −0.857)	0.254
R^2^	0.223			
Adjusted R^2^	0.208			

**Table 6:** Practical ability of healthcare workers toward PHEs and associated factors (linear regression model).

Characteristics	B	*p*-Value	95% Confidence	OR
Gender				
Men				
Women	−0.543	0.167	(−1.313, 0.228)	0.581
Working years				
Less than 5 years				
5–9	1.293	0.004	(0.424, 2.161)	3.644
10–19	1.082	0.015	(0.209, 1.954)	2.951
20–29	1.176	0.010	(0.286, 2.067)	3.241
30 years and above	2.081	<0.001	(0.937, 3.225)	8.012
Highest education				
Junior college or below				
Undergraduate	−0.989	0.001	(−1.596, −0.381)	0.372
Graduate	−1.735	0.049	(−3.461, −0.010)	0.176
Profession				
Doctor				
Nurse	0.783	0.049	(0.003, 1.563)	2.188
Public health personnel	−0.707	0.149	(−1.667, 0.253)	0.493
Pharmacist	−1.797	0.007	(−3.095, −0.498)	0.166
Inspector	0.603	0.378	(−0.740, 1.947)	1.828
Other healthcare workers	−0.182	0.765	(−1.380, 1.016)	0.834
Institutions				
Community health center				
Township health center	0.350	0.248	(−0.245, 0.944)	1.419
Educational activities				
Yes				
No	−1.265	0.001	(−2.014, −0.516)	0.282
Drill activities				
Yes				
No	−1.080	0.004	(−1.822, −0.337)	0.340
Emergency management				
Yes				
No	−1.751	<0.001	(−2.412, −1.089)	0.174
R^2^	0.225			
Adjusted R^2^	0.209			

**Table 7:** Pearson correlation analysis amongst attitude, knowledge, and practical ability.

Dimension	Attitude	Knowledge	Practical Ability
Attitude	1		
Knowledge	0.465 ***	1	
Practical ability	0.458 ***	0.842 ***	1

Note: *** *p* < 0.001.

## Data Availability

The data presented in the study are available upon request from the corresponding authors.

## Supplementary Materials

The following supporting information can be downloaded at: https://www.mdpi.com/article/10.3390/ijerph191912204/s1, Table S1: Knowledge of healthcare workers in PHC institutions towards PHEs; Table S2: Practical ability of healthcare workers in PHC institutions towards PHEs.
