# Supplementary material for: Attitudes and Response Capacities for Public Health Emergencies of Healthcare Workers in Primary Healthcare Institutions: A Cross-Sectional Investigation Conducted in Wuhan, China, in 2020
Source: Int J Environ Res Public Health. 2022 Sep 26;19(19):12204. doi: 10.3390/ijerph191912204 (PMC9564888; doi:10.3390/ijerph191912204)
Supplement: Supplementary file 1 [file ijerph-19-12204-s001.zip › ijerph-1912011-supplementary.pdf]

Supplementary Materials:

Table S1. Knowledge of healthcare workers in PHC institutions towards PHEs

Items	Mean	SD
1. I am knowledgeable about the prevention of PHEs.	4.30	0.861
2. I am knowledgeable about diagnosing and recognising differences in PHEs.	4.23	0.876
3. I am aware of the registration and reporting process for PHEs.	4.23	0.945
4. I am familiar with the treatment and management of patients and suspected patients in PHEs.	4.19	0.909

Table S2. Practical ability of healthcare workers in PHC institutions towards PHEs

Items	Mean	SD
1. I consider myself well prepared to participate in PHEs.	4.17	0.940
2. In the case of PHEs, I consider myself well prepared to participate in emergency management and rescue-related work.	4.17	0.953
3. In the case of PHEs, I can do well in daily related work.	4.21	0.924
4. In the case of PHEs, I can provide health assessments for patients and residents.	4.17	0.937
5. In the case of PHEs, I can provide psychological counselling for patients and residents.	4.15	0.979
